# Association between statin intensity and femoropopliteal stent primary patency in peripheral arterial disease

**DOI:** 10.1186/s42155-024-00472-4

**Published:** 2024-08-03

**Authors:** Elisabeth R. Seyferth, Helen Song, Ansar Z. Vance, Timothy W. I. Clark

**Affiliations:** 1 Section of Interventional Radiology, Department of Radiology, University of Pennsylvania Perelman School of Medicine, Philadelphia, PA USA; 2 University of Pennsylvania Perelman School of Medicine, Penn Presbyterian Medical Center, Philadelphia, PA 19104 USA

**Keywords:** Peripheral arterial disease, Femoropopliteal stent, Restenosis, Statin

## Abstract

**Background:**

Statins are widely used in coronary and peripheral arterial disease, but their impact on patency of stents placed for peripheral arterial disease is not well-studied. The purpose of this study was to evaluate femoropopliteal stent primary patency according to statin intensity at the time of stent placement and compare this effect to other covariates that may influence stent patency.

**Materials and methods:**

A retrospective review identified 278 discrete femoropopliteal stent constructs placed in 216 patients over a 10-year period; Rutherford categories were 2 (3.6%), 3 (12.9%), 4 (21.2%), 5 (49.6%), and 6 (12.6%). Stent locations were common femoral (1.8%), common femoral/superficial femoral (0.7%), superficial femoral (50.7%), superficial femoral/popliteal (32.7%) and popliteal (14.0%) arteries; 63.3% of stents were paclitaxel-eluting. Primary patency of each stent construct was determined with duplex ultrasound, angiography, or computed tomographic angiography. Greater than 50% restenosis or stent occlusion was considered loss of patency. Cox proportional hazard and Kaplan–Meier modeling were used to assess the effect of statin use and additional covariates on stent patency.

**Results:**

Patients on any statin at the time of stent placement were half as likely to undergo loss of primary unassisted patency as patients on no statin therapy (hazard ratio, 0.53; 95% confidence interval, 0.19–0.87; *P* = .004). Moderate/high intensity statin therapy conferred 17 additional months of median stent patency compared to the no statin group. Antiplatelet therapy, anticoagulant therapy, drug-eluting stents (versus bare metal or covered stents), and Rutherford class were not predictive of stent patency (*P* = 0.52, 0.85, 0.58, and 0.82, respectively).

**Conclusion:**

Use of statin therapy at the time of femoropopliteal stent placement was the most predictive examined variable influencing primary unassisted patency.

## Background

Peripheral arterial disease (PAD) is a highly morbid and prevalent disease, affecting at least 8.5 million adults in the United States and more than 230 million adults worldwide [1]. Endovascular therapy of the femoral and popliteal arteries with stent placement is utilized to treat multifocal stenoses and occlusions of these segments and is associated with lower risk of periprocedural complications compared to open surgical treatment [2]. However, stent restenosis and occlusion limit the durability of endovascular treatment, particularly in complex Trans-Atlantic Inter-Society Consensus (TASC) II class C and D lesions [2–5].

Statins are a mainstay of treatment for patients with atherosclerotic disease [6–8]. In addition to reducing low density lipoprotein levels, statins slow progression of intimal-media thickening and attenuate inflammatory changes implicated in cardiovascular disease [9, 10]. However, they remain underutilized in patients with PAD [11, 12] despite societal consensus guidelines recommending their use [13]. The Heart Protection Study, the largest randomized controlled trial to evaluate the effects of statins in PAD patients, demonstrated that the use of statins reduced the rate of first major vascular events including myocardial infarction, cerebrovascular accidents, and all revascularization procedures in this population with a relative risk reduction of 22% [14]. Later studies have shown that statin use reduces limb loss and mortality in PAD, including in a dose-dependent manner in one study [15–17]. Therefore, we hypothesized that statin use increases patency of stents placed for PAD. The purpose of this study was to evaluate femoropopliteal stent patency according to statin intensity at the time of stent placement and compare this effect to other covariates that may influence stent patency.

## Materials and methods

### Study population and data collection

This was a retrospective study of patients undergoing femoropopliteal stent placement for PAD in the interventional radiology department of a single academic institution. Institutional review board approval and a waiver of informed consent were obtained. The procedural database was searched for patients who underwent stenting from January 2012 to June 2023. Five patients who had had prior bypass involving part of the femoropopliteal segment were excluded. After exclusions, 278 femoropopliteal segments underwent stent placement in 216 patients during this period. These 278 stent constructs consisted of one or more overlapping nitinol stents. Patients with long-segment chronic total occlusions involving the origin of the superficial artery extending to the proximal (P1) segment of the popliteal artery might have undergone placement of two or more overlapping stents, however this construct for the purposes of analysis was considered a single stent. When stenotic arterial segments did not require a stent following angioplasty but a stent was needed proximal and distal to these segments, or during separate encounters due to progression of PAD, then these were analyzed as separate stent constructs.

Demographic and comorbid variables collected for each patient included age, sex, body mass index (BMI), smoking history including total pack-years, diabetes mellitus, hypertension, hyperlipidemia, levels of hemoglobin A1c and cholesterol, antiplatelet use, anticoagulant use, statin use, Rutherford category, stent location, and use of a paclitaxel-eluting stent. Demographics of these patients are summarized in Table 1. Mean patient age was 70.3 years (standard deviation 10.7, range 39–94), and 150 patients were male and 128 female. Rutherford categories were 2 (3.6%), 3 (12.9%), 4 (21.2%), 5 (49.6%), and 6 (12.6%).

**Table 1:** Baseline characteristics of patients who underwent stent placement

	**Overall** (*n* = 278)	**No statin** (*n* = 72)	**Low intensity statin** (*n* = 18)	**Moderate intensity statin** (*n* = 78)	**High intensity statin** (*n* = 105)
Female sex	128 (46.0%)	34 (47.2%)	7 (38.9%)	44 (56.4%)	42 (40.0%)
Mean age (SD), years	70.3 (10.7)	71.3 (11.5)	65.9 (12.1)	69.7 (10.9)	70.9 (9.5)
Mean BMI (SD), kg/m^2^	28.0 (6.0)	27.3 (6.0)	25.9 (3.6)	28.8 (6.5)	28.0 (5.9)
Comorbidities					
Current smoker	53 (19.1%)	16 (22.2%)	5 (27.8%)	16 (20.5%)	14 (13.3%)
Former smoker	163 (58.6%)	40 (55.6%)	9 (50.0%)	48 (61.5%)	63 (60.0%)
Diabetes	172 (61.9%)	36 (50.0%)	15 (83.3%)	52 (66.7%)	66 (62.9%)
Hypertension	224 (80.6%)	50 (69.4%)	14 (77.8%)	64 (82.1%)	91 (86.7%)
Hyperlipidemia	157 (56.5%)	23 (31.9%)	13 (72.2%)	46 (59.0%)	73 (69.5%)
Rutherford classification					
2	10 (3.6%)	3 (4.2%)	0	3 (3.8%)	4 (3.8%)
3	36 (12.9%)	7 (9.7%)	3 (16.7%)	14 (17.9%)	12 (11.4%)
4	59 (21.2%)	13 (18.1%)	1 (5.6%)	22 (28.2%)	21 (20.0%)
5	138 (49.6%)	41 (56.9%)	11 (61.1%)	33 (42.3%)	50 (47.6%)
6	35 (12.6%)	8 (11.1%)	3 (16.7%)	6 (7.7%)	18 (17.1%)
Antiplatelet therapy	244 (87.8%)	54 (75.0%)	17 (94.4%)	72 (92.3%)	96 (91.4%)
Anticoagulation therapy	59 (21.2%)	15 (20.8%)	5 (27.8%)	17 (21.8%)	22 (21.0%)
Stent location					
Common femoral (CFA)	5 (1.8%)	1 (1.4%)	0	3 (3.8%)	1 (1.0%)
Superficial femoral (SFA)	141 (50.7%)	37 (51.4%)	9 (50.0%)	42 (53.8%)	50 (47.6%)
Popliteal	39 (14.0%)	9 (12.5%)	2 (11.1%)	10 (12.8%)	18 (17.1%)
CFA-SFA	2 (0.7%)	0	0	1 (1.3%)	1 (1.0%)
SFA-popliteal	91 (32.7%)	25 (34.7%)	7 (38.9%)	22 (28.2%)	35 (33.3%)
Stent type					
Drug-eluting	176 (63.3%)	44 (61.1%)	9 (50.0%)	47 (60.3%)	73 (69.5%)
Non-drug-eluting	102 (36.7%)	28 (38.9%)	9 (50.0%)	31 (39.7%)	32 (30.5%)

Following stent placement, patients were seen in the interventional radiology clinic at 1 month, 3 months, 6 months and 1 year with duplex ultrasound performed at each encounter. Thereafter, patients were seen at yearly intervals unless return of rest pain, new tissue loss or return of disabling claudication occurred at an earlier time interval. Primary unassisted patency of each stent construct was determined with duplex ultrasound, computed tomographic angiography, and conventional angiography, alone or in combination; greater than 50% restenosis or stent occlusion was considered loss of patency [18]. Stents were censored from the analysis at the time of final imaging if they remained patent at that time. Statin intensity at the time of stent placement was categorized by American Heart Association (AHA) guidelines [7].

### Procedural information

**Fig. 1:**
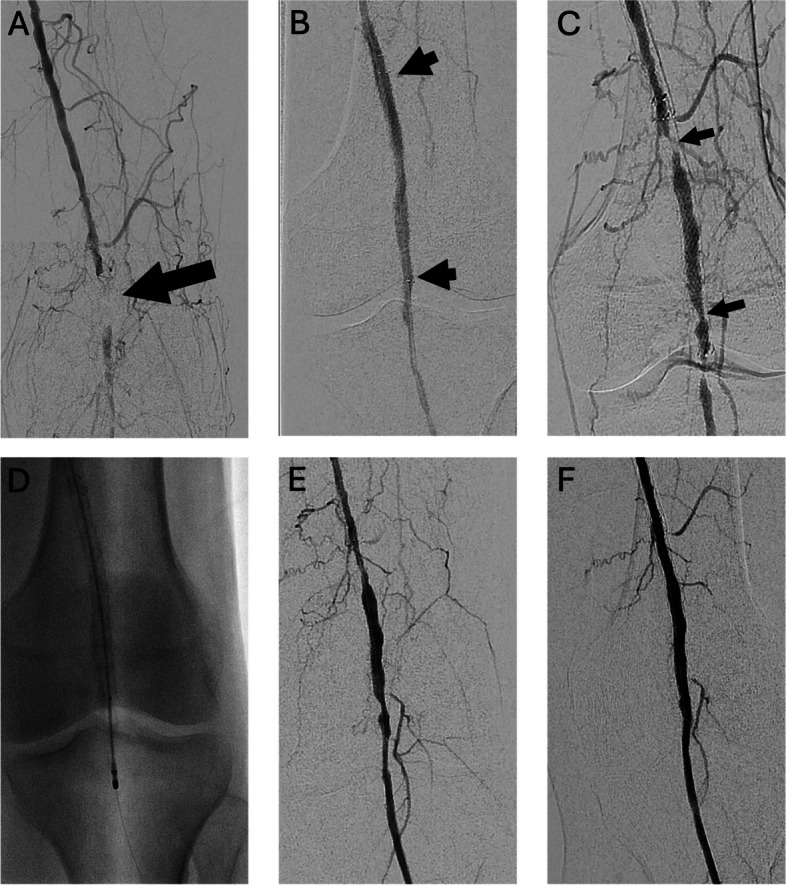
Statin intolerant patient with early loss of stent patency 78-year old male with Rutherford 5 disease and nonhealing left 2nd and 3rd toe wounds. The patient refused statins due to myalgias and was not a candidate for PCSK-9 inhibitors. **A** Initial angiogram showing popliteal artery occlusion (arrow). **B** Angiogram following placement of 6 mm × 10 cm paclitaxel-eluting stent. **C** Angiogram 7 months later after return of rest pain and new forefoot ulcers showing areas of significant in-stent restenosis (arrows), reflecting loss of primary unassisted patency. **D** Fluoroscopic image during thrombectomy (Rotarex, Becton Dickinson, Franklin Lakes, NJ). **E** Post thrombectomy angiogram. **F** Completion angiogram following angioplasty with a 6 mm paclitaxel-eluting balloon

**Fig. 2:**
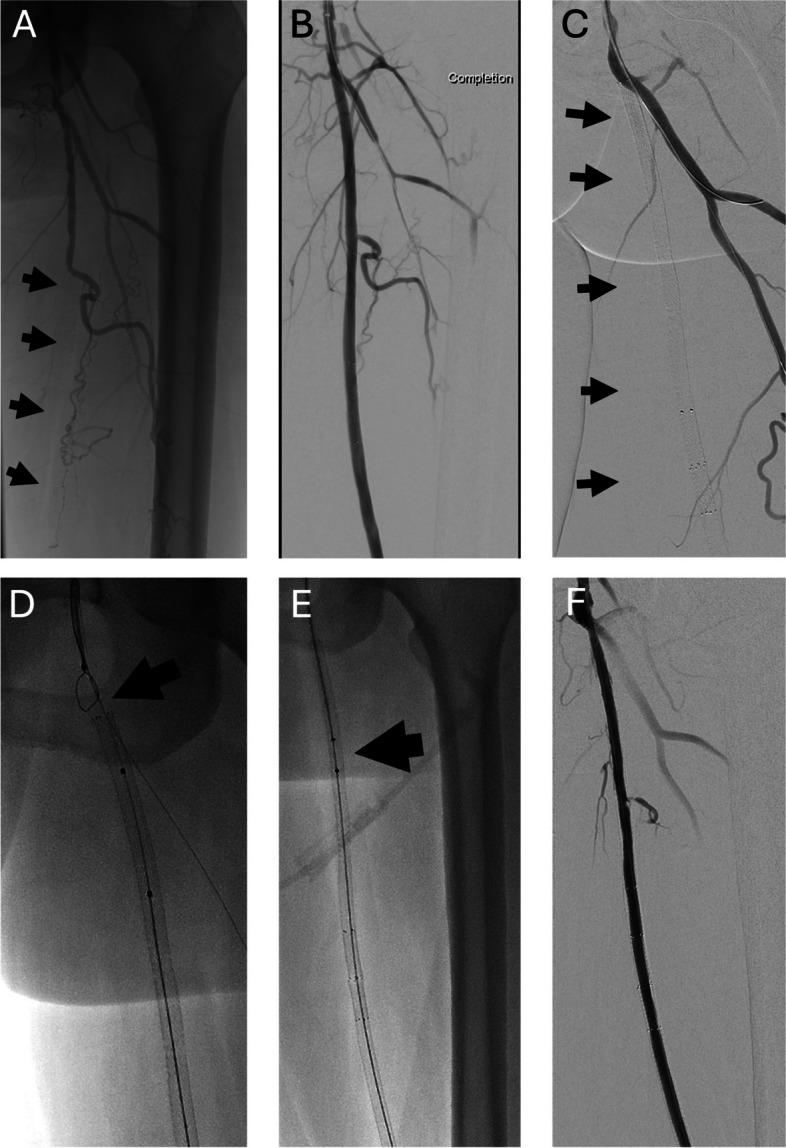
Patient on high intensity statin and sustained stent patency 52-year old woman with lifestyle-disabling claudication without improvement following supervised exercise therapy. **A** Initial angiogram showing long segment chronic total occlusion (CTO) of left superficial femoral artery. **B** Completion angiogram following subintimal recanalization and bare-metal stenting. **C** Initial angiogram after return of severe claudication 73 months later showing occlusion of SFA stent construct (arrows). **D** Crossing of stent occlusion required retrograde pedal access and guidewire rendezvous in the common femoral artery (arrow). **E** Pharmacomechanical thrombectomy of stent construct using 12.5 mg of tissue plasminogen activator and 6 F AngioJet (Boston Scientific, Marlborough, MA) thrombectomy device. **F** Completion angiogram following thrombectomy and 6 mm balloon angioplasty

Antegrade common femoral artery access was used in 92 cases (33.1%), contralateral common femoral access in 170 (61.2%), transtibial/transpedal in 9 (3.2%), and combined contralateral femoral and transpedal in 7 (2.5%). After diagnostic angiography, balloon angioplasty was performed at the stenotic segment followed by stent deployment and post-dilation. Decision to place a stent was per the discretion of the attending interventional radiologist, but typically in cases in which there was persistent stenosis > 50% following initial balloon angioplasty, flow-limiting dissection not responsive to prolonged balloon angioplasty, or in a segment in which a chronic total occlusion was crossed subintimally. Stent locations were as follows: 5 common femoral (1.8%), 2 common femoral/superficial femoral (0.7%), 141 superficial femoral (50.7%), 91 superficial femoral/popliteal (32.7%), and 39 popliteal (14.0%) artery. Most (63.3%) stents were paclitaxel-eluting. Examples of in-stent restenosis among patients receiving low-intensity statin therapy and high intensity statin therapy are shown in Figs. 1 and 2.

### Data analysis

**Fig. 3:**
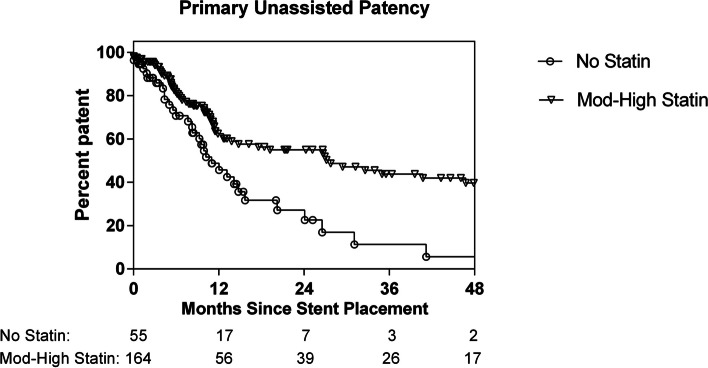
Kaplan–Meier curve comparing loss of primary femoropopliteal segment stent patency in patients on moderate or high intensity statin therapy versus no statin therapy (log-rank test, *P* = .0006)

**Fig. 4:**
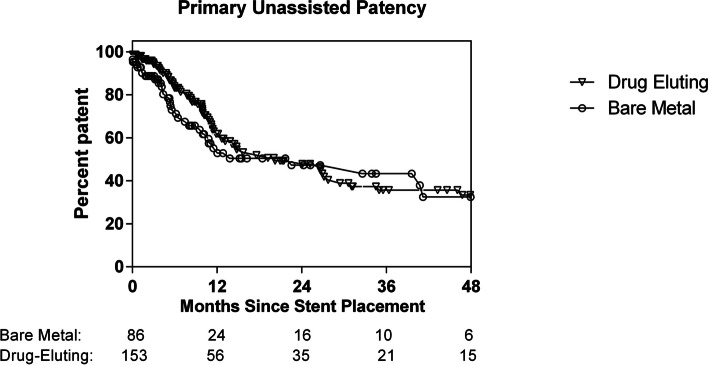
Kaplan–Meier curve comparing loss of primary femoropopliteal segment stent patency between paclitaxel-eluting stents and bare metal stents (log-rank test, *P* = .09)

Statin use, age, sex, BMI of 30 kg/m^2^ or greater, smoking history and total pack-years, the diagnosis of diabetes, hypertension, hyperlipidemia, coronary artery disease (CAD), and end stage renal disease (ESRD), hemodialysis use, hemoglobin A1c and cholesterol levels, Rutherford score, antiplatelet and anticoagulation medication use, popliteal stent placement, and drug-eluting stent use were analyzed as covariates with univariate Cox proportional hazards modeling to assess for potential effects on stent patency. Multivariate Cox proportional hazards modeling including the statistically significant univariate variables was performed. A Kaplan–Meier analysis was used to compare the duration of primary unassisted patency between the moderate and high intensity statin group and the no statin group (Fig. 3). Kaplan-Meier analysis was also used to compare primary unassisted patency between bare and drug-eluting stents (Fig. 4), irrespective of statin intensity. The Mantel-Cox log-rank test was used to evaluate for a difference in median duration of primary patency based on statin use. Statistical analysis was carried out using Stata version 14.1 (Stata Corporation, College Station, TX). A *P*-value of < 0.05 was considered statistically significant.

## Results

Of the 278 femoropopliteal stents placed, 38 had no imaging follow up from our institution and were excluded from further analysis. 42.5% of stents (102 of 240) lost primary patency in the study period. Median time to loss of primary patency was 8.9 months (range: 0 days to 9.8 years). 38 lost patency within 6 months of placement (37.2%), 33 between 6 months and 1 year (32.4%), 12 between 1 and 2 years (11.8%), and 19 in greater than two years (18.6%). Among stents that had imaging follow up at one year, primary patency was 53.3% (80 of 150). Among those that had follow up at two years, primary patency was 37.1% (49 of 132). Of the 138 stents that remained patent on imaging, the median duration of imaging follow up was 6.6 months (range: 1 day to 7.4 years). 90 had less than a year of imaging follow up but remained patent to that point, and 108 had less than two years of follow up but remained patent to that point.

Patients who were on moderate or high intensity statin therapy at the time of stent placement were half as likely to experience loss of primary patency by univariate Cox regression analysis (HR, 0.50; 95% CI, 0.18–0.82, *P* = 0.003) (Table 2). Patients who were taking a statin of any intensity at the time of stenting were also half as likely to experience loss of primary patency (HR, 0.53; 95% CI, 0.19–0.87; *P* = 0.004). The use of a moderate or high intensity statin was associated with a median increase in duration of stent patency of 16.7 months, with a median time to loss of primary patency of 27.8 months in the statin group and 11.0 months in the group not taking a statin (log-rank test, *P* = 0.0006; Fig. 1). The presence of hypertension (HR, 0.56; 95% CI, 0.22–0.90, *P* = 0.02), male sex (female sex HR, 1.60; 95% CI, 0.53–2.67, *P* = 0.02), and CAD (HR, 0.60; 95% CI, 0.22–0.98, *P* = 0.03) were also associated with improved primary patency. In multivariate Cox regression analysis including moderate or high intensity statin use, sex, hypertension, and CAD, only hypertension remained significant (HR, 0.53; 95% CI, 0.24–0.82, *P* = 0.04) (Table 3). To explore whether this finding was attributable to increased statin use among patients with CAD, multivariate modeling was performed after excluding CAD (Table 4). This modeling showed similar effects of moderate and high intensity statin therapy on lowering risk of loss of primary patency compared to univariate analysis (HR, 0.54; 95% CI, 0.20–0.88, *P* = 0.008), however hypertension was no longer associated with improved primary patency. Female sex was associated with lower primary patency in this model (HR, 1.91; 95% CI, 0.67–3.15, *P* = 0.003), as was observed in univariate analysis.

**Table 2:** Factors affecting loss of femoropopliteal stent patency by univariate Cox proportional hazards analysis

Factor	Hazard ratio (95% CI)	*P* value
High intensity statin use	0.58 (0.35)	**0.035**
Moderate or high intensity statin use	0.50 (0.32)	**0.003**
Any statin use	0.53 (0.34)	**0.004**
Female sex	1.60 (1.07)	**0.022**
Age	1.00 (0.98)	0.715
BMI > 30	1.19 (0.78)	0.418
Smoking history	1.02 (0.79)	0.887
Pack-years of smoking	1.01 (0.99)	0.394
Diabetes	1.07 (0.70)	0.755
Hypertension	0.56 (0.34)	**0.024**
Hyperlipidemia	0.68 (0.44)	0.075
Coronary artery disease	0.60 (0.38)	**0.028**
End stage renal disease	1.03 (0.55)	0.917
Hemodialysis	1.04 (0.56)	0.902
Hemoglobin a1c	1.13 (0.72)	0.585
Total cholesterol	1.00 (0.99)	0.428
Non-HDL cholesterol	1.00 (0.99)	0.272
Triglycerides	1.00 (1.00)	0.821
Rutherford score	1.06 (0.65)	0.823
Antiplatelet medication	0.81 (0.42)	0.522
Anticoagulation medication	1.05 (0.63)	0.849
Placement of popliteal stent	1.30 (0.86)	0.208
Use of drug-eluting stent	0.89 (0.58)	0.579

Bold font indicates *P* < .05

**Table 3:** Factors affecting loss of femoropopliteal stent patency by multivariate Cox proportional hazards analysis

Factor	Hazard Ratio (95% CI)	*P* value
Moderate or high intensity statin use	0.62 (0.37)	0.072
Female sex	1.50 (0.90)	0.117
Hypertension	0.53 (0.29)	**0.044**
Coronary artery disease	0.74 (0.44)	0.262

Bold font indicates *P* < .05

**Table 4:** Factors affecting loss of femoropopliteal stent patency by multivariate Cox proportional hazards analysis after exclusion of CAD

Factor	Hazard Ratio (95% CI)	*P* value
Moderate or high intensity statin use	0.54 (0.34)	**0.008**
Female sex	1.91 (1.24)	**0.003**
Hypertension	0.64 (0.37)	0.102

Bold font indicates *P* < .05

Although drug-eluting stents conferred no significant benefit in primary unassisted patency in univariate Cox models, Kaplan–Meier estimates showed somewhat higher patency in the first 12 months. The 3-, 6-, and 12-month primary patency (± standard error of mean) among paclitaxel-eluting stents was 95.0 ± 2.0, 83.2 ± 3.4, and 61.9 ± 4.8%, compared to 88.8 ± 3.5, 73.0 ± 5.6, and 55.2 ± 6.7% (*P* < 0.05) among bare metal stents. Thereafter, the patency rates overlapped through 48 months (log-rank test, *P* = 0.09) (Fig. 2).

## Discussion

This study demonstrates a significant association of primary patency of femoropopliteal stents with statin therapy. The use of any statin, regardless of statin intensity, was associated with a median increase in duration of stent patency of 17 months. In addition to lowering lipid levels, statins have been shown to modulate inflammatory pathways in cardiovascular disease, including inhibition of major histocompatibility complex class II induction in human smooth muscle cells and fibroblasts [19]. Further, statins are known to change the expression of factors involved in fibrinolysis, including plasminogen activator inhibitor-1 [20]. Together the modulation of these pathways may reduce vascular smooth muscle cell and fibroblast activation that play a central role in in-stent restenosis and/or stent thrombosis [9, 21, 22]. Prior studies have demonstrated that statin therapy is associated with lower rates of major vascular events including revascularization procedures, greater amputation-free survival, and lower mortality rates in patients with PAD [14–17, 23, 24]. Statin use has also been found to correlate directly to improved infrainguinal graft patency [25, 26]. However, a paucity of data exists which addresses association between statins and stent primary patency [27–29].

A diagnosis of CAD, hypertension, and male sex were also associated with lower risk of primary patency loss in univariate analyses. CAD may be associated with a lower risk of patency loss in this analysis because statin use was higher in CAD patients (81.7% of patients with CAD were taking a statin, while 67.7% of patients without CAD were taking one). In line with this hypothesis, a multivariate analysis that included hypertension, CAD, sex, and statin use found that only hypertension remained a significant predictor, but when CAD was excluded statin use was again significant. The reason for the association of stent patency with hypertension in the univariate analysis is unclear and has not been consistently shown in the literature; it may relate to the diagnosis and treatment of hypertension in the CAD versus non-CAD population given that it was no longer significant in the multivariate analysis once CAD was excluded. It is also possible that some of the patients in this study who did not carry the diagnosis did have hypertension but were untreated, while the patients with diagnosed hypertension were more adequately treated and therefore less likely to experience loss of stent patency. Finally, the association of female sex with greater risk of patency loss is consistent with prior work, including a post hoc analysis of the IN.PACT SFA trial that demonstrated a trend of female sex correlating with worse clinical outcomes after endovascular treatment of femoropopliteal disease [30]; further work is needed to more fully characterize gender differences in endovascular therapies for PAD.

Interestingly, additional variables that have been previously associated with stent restenosis and the progression of peripheral arterial disease in general were not predictive of the duration of stent patency in this study. These include stent placement in the popliteal artery, factors such as current smoking and use of antiplatelet therapy, and notably, use of a drug-eluting stent [31–35]. There are fundamental differences between the clinical trials that have demonstrated differences in patency between drug-eluting and bare metal stents and this study, which may explain the lack of effect of stent type on patency in this population beyond the modest benefit realized with paclitaxel stents within the first 12 months following implantation. This was a real-world patient experience with many complex lesions including chronic total occlusions, as well as inclusion of patients without strict requirements for target vessel diameter, target lesion length, and other specific lesion factors [5, 36]. ESRD status also did not confer a higher risk of stent patency loss, despite previous studies showing that it correlates to major adverse limb events among chronic limb ischemia patients; tibial occlusive disease may be a larger driver of limb loss in ESRD patients rather than femoropopliteal disease [37].

The current study observed a one-year primary patency rate of 53% and a two-year patency rate of 37%. These results, which reflect a broad spectrum of anatomic location and comorbid patient factors, are within the range of recent studies reporting primary patency rates of 43–85% at one year and 37–71% at two years [3, 4, 34, 36, 38, 39]. The varying patency rates across the literature may be due to differences in patient population, lesion complexity, and exclusion criteria; for example, many studies exclude P2 and/or P3 popliteal lesions [3, 32, 36].

The use of statins in this population of patients with symptomatic PAD was consistent with prior studies. Despite long-standing recommendations for patients who are at high risk for atherosclerotic cardiovascular disease to be prescribed a high intensity statin—which includes patients with a history of atherosclerotic PAD [8, 13]—26.4% of patients were not taking a statin at the time of initial intervention and 6.6% were on a low intensity statin. Especially given the association of statin use with stent patency in this study, these rates confirm the need to continue to increase statin use in these patients. Encouragingly, statin use in this study’s population has improved in recent years due to an active effort in IR clinic visits to increase statin adoption and compliance—in the last 5 years, the percent of patients not taking a statin declined to 19.9%, and further declined to 15.8% in the last two years.

This study has several limitations. First, it is a retrospective single center study with unmatched patient characteristics within each level of statin therapy. Some patients had limited follow up, which may skew the reported patency rates given that follow up with imaging was required to confirm patency over time. In addition, selected situations where individual non-overlapping stents were present in the same patient were treated as distinct data points in the statistical analysis. There were also several patients who changed statin therapy after the stent was placed—given the overall low numbers of such patients, they were categorized under the statin intensity they were receiving at the time of stent placement. Some variables that have been previously shown to correlate with duration of stent patency, such as stent placement in an area of chronic total occlusion, smaller vessel diameter, and greater stent length, were not assessed in this study [31, 32]. We also recorded antiplatelet therapy as a dichotomous variable and thus did not analyze the potential effect of single versus dual antiplatelet therapy. Finally, the study hypothesis was limited to examining just the effects of statins and other covariates on primary unassisted patency. Those patients who lost primary patency returned to undergo additional interventions to restore patency, but the potential effects of statins on primary assisted and secondary stent patency were not examined.

## Conclusions

In conclusion, this study demonstrates an association between statin therapy and femoropopliteal stent primary patency, providing additional support for the importance of statin therapy in patients with peripheral arterial disease. As physicians treating this population, interventional radiologists are in an ideal position to ensure that PAD patients are appropriately prescribed statins.

## Data Availability

The datasets used and/or analyzed during the current study are available from the corresponding author on reasonable request.

## Abbreviations

PAD: Peripheral arterial disease
TASC: Trans-Atlantic Inter-Society Consensus
BMI: Body mass index
SD: Standard deviation
CFA: Common femoral artery
SFA: Superficial femoral artery
AHA: American Heart Association
CAD: Coronary artery disease
ESRD: End stage renal disease
HR: Hazard ratio
CI: Confidence interval
